# Affinity Is an Important Determinant of the Anti-Trypanosome Activity of Nanobodies

**DOI:** 10.1371/journal.pntd.0001902

**Published:** 2012-11-15

**Authors:** Guy Caljon, Benoît Stijlemans, Dirk Saerens, Jan Van Den Abbeele, Serge Muyldermans, Stefan Magez, Patrick De Baetselier

**Affiliations:** 1 Department of Biomedical Sciences, Unit of Veterinary Protozoology, Institute of Tropical Medicine Antwerp, Antwerp, Belgium; 2 Research Unit of Cellular and Molecular Immunology, Vrije Universiteit Brussel, Brussels, Belgium; 3 Laboratory of Myeloid Cell Immunology, VIB, Brussels, Belgium; 4 Department of Structural Biology, VIB, Brussels, Belgium; Foundation for Innovative New Diagnostics (FIND), Switzerland

## Abstract

**Background:**

The discovery of Nanobodies (Nbs) with a direct toxic activity against African trypanosomes is a recent advancement towards a new strategy against these extracellular parasites. The anti-trypanosomal activity relies on perturbing the highly active recycling of the Variant-specific Surface Glycoprotein (VSG) that occurs in the parasite's flagellar pocket.

**Methodology/Principal Findings:**

Here we expand the existing panel of Nbs with anti-*Trypanosoma brucei* potential and identify four categories based on their epitope specificity. We modified the binding properties of previously identified Nanobodies Nb_An05 and Nb_An33 by site-directed mutagenesis in the paratope and found this to strongly affect trypanotoxicity despite retention of antigen-targeting properties. Affinity measurements for all identified anti-trypanosomal Nbs reveal a strong correlation between trypanotoxicity and affinity (K_D_), suggesting that it is a crucial determinant for this activity. Half maximal effective (50%) affinity of 57 nM was calculated from the non-linear dose-response curves. In line with these observations, Nb humanizing mutations only preserved the trypanotoxic activity if the K_D_ remained unaffected.

**Conclusions/Significance:**

This study reveals that the binding properties of Nanobodies need to be compatible with achieving an occupancy of >95% saturation of the parasite surface VSG in order to exert an anti-trypanosomal activity. As such, Nb-based approaches directed against the VSG target would require binding to an accessible, conserved epitope with high affinity.

## Author Summary

Nanobodies, antigen binding fragments derived from a non-conventional class of antibodies in camelids, were previously shown to exert a direct activity against African trypanosomes without the need of a toxin. Their mode-of-action relies on interference with the highly active recycling of the Variant-specific Surface Glycoprotein (VSG) that occurs in the flagellar pocket of the parasite. By expanding the panel of anti-trypanosomal Nanobodies and by modification of their binding properties through site-directed mutagenesis, we have been able to show a strong correlation between their trypanotoxic activity and affinity for the cognate antigen. From these studies it was calculated that the parasite surface saturation needs to exceed 95% in order to achieve this anti-trypanosomal effect of Nanobodies, which can be considered as a critical cut-off value for future Nanobody-based or other small molecule drug approaches against the VSG target.

## Introduction

African trypanosomes are extracellular protozoan parasites that are responsible for human sleeping sickness and trypanosomiasis in livestock. Studies of *Trypanosoma brucei* infections in mice have shown that these parasites cope with the host adaptive immune system through suppression of T and B cell responses [1]–[4] and antigenic variation of Variant-specific Surface Glycoprotein (VSG), the most abundant GPI-anchored membrane protein [5], [6]. Due to stochastic genetic alterations, *T. brucei* changes its antigenic VSG-coat by which antibody responses mounted by the host are not protective throughout infection [7], [8]. Moreover, the molecular organization of 5×10^6^–10^7^ VSG copies per cell as densely packed dimers [9], results in shielding of invariant surface epitopes. Recently, some trypanosome-specific Nanobodies (Nbs) were shown to exert an *in vitro* and *in vivo* activity against the *T. brucei* AnTat1.1 variant antigen type (VAT), independent of the complement activation pathway. Nbs are antigen-binding VHH fragments of approximately 15 kDa derived from Heavy-chain Antibodies that are present in *Camelidae* [10] and can be selected through phage-display technology and panning [11], [12]. Experimental data suggested that the toxicity of the Nanobody depends on interference with the highly active VSG-recycling mechanism. In normal circumstances, the bloodstream form *T. brucei* is able to turn-over its total surface-exposed VSG pool within 12 minutes through uptake in clathrin-coated vesicles and exocytosis in the flagellar pocket area [13]. This VSG recycling would allow antibody shedding and protection against destructive host immune responses by internalization of immune complexes [14], [15]. Illustrating that Nanobodies interfere with this cell trafficking mechanism, Nanobody-mediated trypanotoxicity is associated with disturbances in endo/exocytosis and a pronounced expansion of the flagellar pocket [16]. Despite these phenotypic characteristics of toxicity, the exact mode-of-action of the available AnTat1.1 VSG-specific Nanobodies remains to be elucidated. Nevertheless, it is clear that an interference with the early onset of endocytosis through the flagellar pocket is involved.

Here we report on the identification of a larger panel of *T. brucei* AnTat1.1 VSG-specific trypanotoxic Nanobodies from a new phagemid library and document that high affinity (nanomolar range) for VSG is a common characteristic of all identified trypanotoxic Nanobodies. In addition, we show that affinity reduction by randomization of the amino acids within the antigen-binding loops of the Nanobody or by mutation of framework residues strongly reduces the trypanotoxic potential despite retention of antigen-binding capacity. Site-directed mutagenesis to introduce humanizing mutations in framework-2 [thus replacing residues that are different between VHHs and human VHs, [17], [18]] is shown to preserve trypanotoxic activity only if the affinity is not drastically reduced. We illustrate that there is a strong correlation between trypanotoxic potential and affinity (K_D_) constants. We further discuss the implications of these observations for Nanobody-based anti-trypanosomal interventions.

## Methods

### Ethics statement

The experiments, maintenance and care of mice complied with the guidelines of the European Convention for the Protection of Vertebrate Animals used for Experimental and other Scientific Purposes (CETS n° 123). Parasites were expanded in in-house bred six to eight weeks old C57B1/6 mice. Mouse care and experimental procedures were performed under approval from the Animal Ethical Committee of the Vrije Universiteit Brussel (Ref. Nr. 08-220-8).

### Alpaca immunization against trypanosomal Variant-specific Surface Glycoprotein (VSG)

Soluble form of the VSG (sVSG) from different *Trypanosoma* species including *T. b. brucei* AnTat1.1, MiTat1.2 and MiTat1.5, *T. b. rhodes*iense Etat1.2R, *T. b. gambiense* LiTat 1.3, *T. evansi* ITMAS 120399A, *T. vivax* 700 and *T. congolense* Tc13 was purified as described elsewhere [19], [20] and stored at −20°C. An alpaca (Diaclone, France) was immunized by weekly subcutaneous injections of at least 200 µg of one of the above VSG antigens in presence of the same volume of Gerbu adjuvant (every week another sVSG for 8 consecutive weeks).

### Generation of an alpaca sVSG-immune library

Four days after the last injection, blood was collected for the isolation of peripheral blood lymphocytes and preparation of cDNA. VHH gene fragments ranging from codons from framework region 1 to framework region 4 were amplified by nested PCR as described elsewhere [21], [22]. Amplicons were unidirectionally ligated using the *Nco*I and *Bst*EII restriction sites of the in-house phagemid vector pMES, derived from pHEN4 [23]. The ligated material was subsequently transformed in *E. coli* TG1 cells and stored at −80°C in LB-medium supplemented with 50% glycerol.

### Generation of a randomized library based on Nanobody 33 (Nb_An33)

Residues in complementarity determining regions (CDR) 1 and 3 of the previously identified Nb_An33 [24], and putatively involved in the recognition of *T. brucei* sVSG, were identified based on the Nanobody crystallographic structure [18]. A Nb_An33 library with randomized codons in the paratope, at five amino acid positions either in CDR1 (Ser^29^, Thr^30^, Tyr^31^, Ser^32^ and Thr^35^) or CDR3 (Val^103^, Arg^104^, Ser^105^, Ile^106^ and Arg^107^) has been generated by PCR using degenerate primers. The codons for the residues of interest were replaced by NNK (K = G or T). Ligation of the amplicons into the pMES phagemid was performed using *Pst*I and *Bst*EII restriction sites. Ligated material from the randomization in CDR1 and CDR3 was pooled and used for transformation as described for the alpaca sVSG immune library (see above).

### Selection of sVSG-specific Nanobodies through panning

The sVSG immune library and Nb_An33 randomized library were expressed on phages after super-infection with M13K07 helper phages. Libraries were enriched by several consecutive rounds of *in vitro* selection on solid-phase coated *T. b. brucei* AnTat1.1 sVSG using a standard procedure [22], [24], [25]. Phages were eluted under alkaline conditions (pH 11.0), followed by an immediate pH neutralization and amplification in *E. coli* TG1 cells [22]. Enrichment of the library for antigen-specificity was assessed using an AnTat1.1 sVSG-specific phage ELISA. Here, phages that are specific for AnTat1.1 sVSG were detected using a horse-radish peroxidase-anti-M13 conjugate (Amersham Biosciences). As soon as enrichment was observed in the phage ELISA, individual colonies of transfected TG1 cells were picked to evaluate the expression of antigen-specific Nanobodies upon induction with 1 mM isopropyl-β-d-thiogalactopyranoside (IPTG). Periplasmic extracts were tested for antigen recognition in ELISA using a peroxidase-conjugated mouse anti-6×His IgG (Serotec).

### Expression and purification of Nanobodies

*T. brucei* specific clones were used to purify plasmid (pMES with the VHH gene of interest). The plasmid constructs were transformed into chemocompetent *E. coli* WK6 (su^−^) cells. Expression and Nanobody purification was similar as described earlier [25]. Nanobodies were eluted from Ni-NTA columns using 0.5 M imidazole/PBS and were subsequently subjected to gelfiltration on a Superdex 75 10/300 GL column (GE Healtcare) with phosphate buffered saline (PBS) as running buffer.

### Surface plasmon resonance

Affinities of newly identified Nanobodies or Nb_An33 mutants were measured by surface plasmon resonance on a BIAcore T100 system. Between 1000 and 1500 RU of soluble AnTat1.1 VSG was coupled onto a CM5 chip (BIAcore) via amine groups according to the manufacturer's descriptions using EDC and NHS as cross-linking agents and ethanolamine to block free esters. For the affinity determination, Nanobody concentrations ranging from 500 to 7.8 nM were added to the antigen-coated chip at a flow-rate of 30 µl/min in HBS buffer [10 mM Hepes (pH 7.5), 150 mM NaCl, 3.5 mM EDTA, 0.005% (v/v) Tween-20)]. The flow cells were regenerated with 10 mM glycine-HCl (pH 1.5). Sensograms were fitted for a 1∶1 binding model with local R_max_ using the BIA-evaluation software version 4.1 (BIAcore), resulting in association and dissociation constants (k_a_ and k_d_) as output from which affinity (K_D_) values were calculated.

Epitope mapping was performed on the same VSG sensor chip by evaluating cumulative or competitive binding onto the antigen with 10-fold higher Nanobody concentrations than the K_D_ using all possible two-by-two Nanobody combinations.

### Flow cytometry and fluorescence microscopic analysis

Binding of the different Nanobody clones was evaluated on live, bloodstream form *T. b. brucei* AnTat1.1 through flow cytometry following a direct or three-step labelling procedure. The direct labeling required conjugation of Nanobodies with Alexa Fluor 488 according to the manufacturer's instructions (Molecular Probes). The three-step labeling procedure relied on the detection of the surface-bound Nanobodies with a mouse anti-6×His IgG and a phycoerythrin-labelled rat anti-mouse IgG. Flow cytometry analyses were performed on a FACS Canto II and histograms were prepared using the FlowJo software (Becton Dickinson, San Jose, CA). Stained trypanosomes were subsequently fixed for 30 minutes using 3% paraformaldehyde and washed five times with 0.1 M HEPES pH 7.4 in order to facilitate analysis by fluorescence microscopy as this procedure stops cell motility and VSG recycling by endocytosis. Cells were stained with DAPI by the use of the Fluoro Gel II mounting medium (Electron Microscopy Sciences).

### Trypanotoxicity experiments

Trypanotoxic activity of Nanobodies was evaluated on *ex vivo* isolated and DEAE52-purified trypanosomes. Parasite cultures were initiated at 10^5^/ml concentrations in 200 µl HMI-9 medium supplemented with 10% decomplemented fetal bovine serum (FBS). Nanobodies were added to the parasites at concentrations of respectively 10, 20, 40 and 60 µg/ml followed by incubation at 37°C in a conditioned atmosphere with 5% CO_2_. Toxicicty was quantified by parasite counting using a Bürker hematocytometer after 18 h incubation. Percentage toxicity was calculated from at least two individual wells and relative to the condition with a non-trypanosome specific Nanobody. An additional buffer control (PBS) was included in the lysis experiments.

### Data and statistical analysis

Correlation between the trypanotoxic potential of the different anti-trypanosome Nanobodies and their affinity constants k_a_, k_d_ and K_D_ was analyzed by the non-parametric Spearman correlation analysis using the GraphPad Prism 5.02. Reported data are the Spearman correlation coefficients (*r*) that vary between +1 and −1 depending on a positive or negative correlation between the variables. Dose response curves were obtained in the same software package by a three parameter dose response non-linear regression with a top/bottom constraint of respectively 100% and 0%. As output of this regression analysis, half maximal effective K_D_, k_a_ and k_d_ values (affinity and kinetic rate constants resulting in 50% trypanotoxicity) are reported as well as the 95% Confidence Intervals (CI). Based on the Michaelis-Menten equation assuming a single binding site and non-cooperative binding, the VSG binding site occupancy (B/B_max_) at steady state for the determined half maximal effective K_D_ values of the Nbs can be determined from the equation:
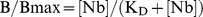



## Results

### Identification of a panel of trypanotoxic Nanobodies

**Figure 1:**
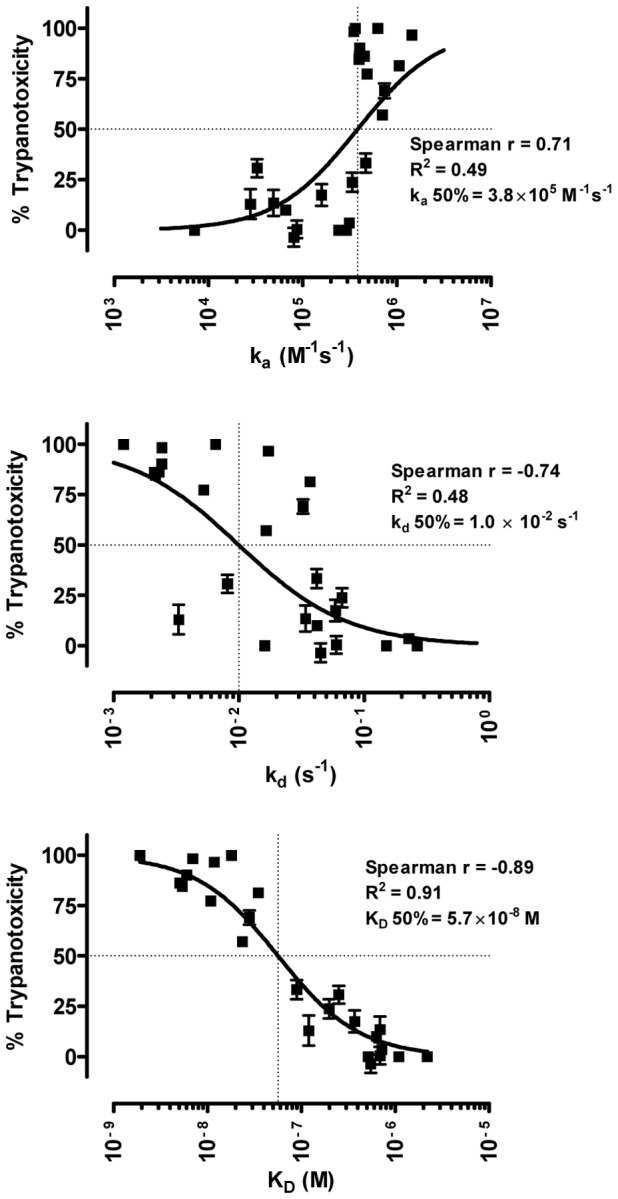
Functional characterization of the newly identified Nanobodies. (**A**) Flow cytometric analysis demonstrating Nanobody binding onto live bloodstream form *T. brucei* AnTat1.1 using a three step labeling with the Nanobody, mouse anti-6×His IgG and phycoerythrin conjugated rat anti-mouse IgG (**B**) Dot plot representation with isoaffinity lines of the individual k_a_, k_d_ and K_D_ affinity constants of the different newly identified anti-*T. brucei* Nbs (**C**) *T. brucei* trypanotoxic activity at 37°C measured after 18 hours with 10 and 20 µg/ml of the different Nbs in HMI-9/10%FCS medium calculated relative to the condition with a non-trypanosome specific Nb and including a buffer (PBS) control.

Retrieval of AnTat1.1 sVSG-specific Nanobodies from the library was performed by four consecutive rounds of *in vitro* selection on solid–phase coated *T. brucei* AnTat1.1 sVSG. This resulted in the identification of eight different novel VHHs (Figure S1) with antigen-binding capacity and sequence variations in the complementarity determining regions: Nb_An-R3.7, Nb_An-R3.68, Nb_An-R3.92, Nb_An-R4.11, Nb_An-R4.29, Nb_An-R4.55, Nb_An-R4.71, Nb_An-R4.80. All eight newly identified Nanobodies were able to bind their epitope on live trypanosomes (Figure 1A) and their affinity constants were determined using surface plasmon resonance (SPR) measurements on a CM5 chip coupled with 1000–1500 RU purified *T. brucei* AnTat1.1 sVSG. Affinities were all in the low nanomolar range (Figure 1B), ranging from 5 to 35 nM [ Nb_An-R3.7 (5 nM), Nb_An-R3.68 (12 nM), Nb_An-R3.92 (11 nM), Nb_An-R4.11 (35 nM), Nb_An-R4.29 (5 nM), Nb_An-R4.55 (5 nM), Nb_An-R4.71 (7 nM), Nb_An-R4.80 (6 nM)] and displayed >80% trypanotoxic activity at 10 µg/ml within 18 hours of incubation in HMI-9/10% FBS medium (Figure 1C). Most potent trypanotoxic Nanobodies were the previously identified Nb_An05 and Nb_An46 [16], resulting in 100% lysis while Nb_An33 exerted more moderate effects, resulting in 65–75% lower parasite counts after overnight incubation.

### Affinity reduction through mutagenesis decreases trypanotoxic potential

Previous randomization of selected tyrosine residues in CDR1 and CDR3 of the wildtype and highly trypanotoxic Nb_An05 resulted in the identification of several paratope-variants with retained trypanosome-binding potential but with reduced trypanotoxicity and affinity that ranged from 120 nM to 2.2 µM [ Nb_An05-02 (2.2 µM), Nb_An05-04 (250 nM), Nb_An05-05 (690 nM), Nb_An05-06 (370 nM), Nb_An05-09 (630 nM), Nb_An05-12 (120 nM), Nb_An05-17 (550 nM), Nb_An05-19 (690 nM); [16]].

**Figure 2:**
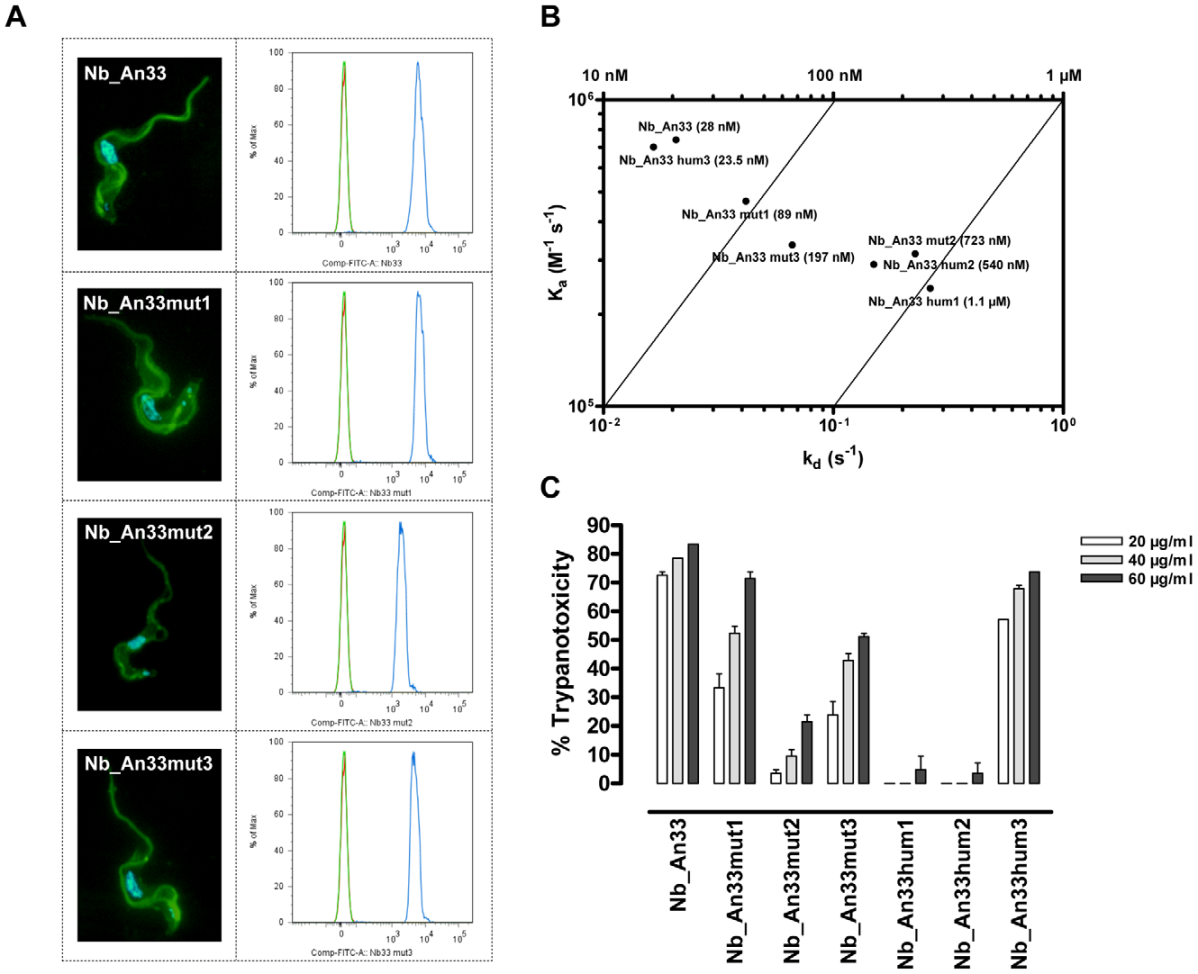
Functional characterization of the Nb_An33 variants. (**A**) Binding studies with ALEXA 488-labeled Nb_An33 and Nb_An33mut1-3 variants onto purified bloodstream form *T. brucei*: fluorescence microscopic images of trypanosomes stained with DAPI and the respective Nanobodies and flow cytometric analysis of trypanosomes incubated without Nanobody (green line), with the different Nb_An33 variants (blue line) or with an ALEXA 488-labeled control Nanobody (red line). (**B**) Dot plot representation with isoaffinity lines of the individual k_a_, k_d_ and K_D_ affinity constants of Nb_An33, the paratope variants (Nb_An33mut1-3) and humanized framework variants (Nb_An33hum1-3). (**C**) Trypanotoxic activity at 37°C measured after 18 hours with 20, 40 and 60 µg/ml of the different Nb_An33 variants in HMI-9/10%FCS medium.

Based on information from the Nb_An33 crystal structure, residues within the Nb_An33 paratope (Ser^29^, Thr^30^, Tyr^31^, Ser^32^ and Thr^35^ in CDR1 or Val^103^, Arg^104^, Ser^105^, Ile^106^ and Arg^107^ in CDR3) were randomized by site-directed mutagenesis, combined with the generation of a phagemid library to allow selection of functional AnTat1.1 sVSG-specific Nb_An33 variants through phage display and panning. Pannings, combined with expression and sequence analysis of individual clones resulted in the selection of three Nb_An33 variants with retained antigen-binding properties. One variant was mutated in CDR1 (Nb_An33mut1: Ser29Arg/Thr30Ala/Tyr31Leu), two variants had substitutions in CDR3 (Nb_An33mut2: Val103Phe/Arg104Val/Ser105Asn/Arg107Asn and Nb_An33mut3: Val103Asn/Arg104Asp/Ser105Cys/Arg107Leu) (Figure S2). Flow cytometry analysis revealed proper binding of all Nb_An33 derived variants to living *T. brucei* (Figure 2A). Comparative epitope mapping of Nb_An33 and the 3 mutants in SPR revealed that the identified variants bind to the same epitope or at least epitopes in a common region as no significant cumulative binding was observed onto the VSG-sensor chip for any Nanobody combination (data not shown). Affinity measurements by surface plasmon resonance revealed significant reduction in affinity of the 3 mutants, i.e. 89, 723 and 197 nM as compared to the wildtype Nb_An33 (K_D_ = 28 nM) (Figure 2B, Table 1). Also on- and off-rate constants were negatively affected to different extends through mutagenesis, reducing the k_a_ from 7.41×10^5^ M^−1^s^−1^ to 4.67×10^5^, 3.14×10^5^ and 3.36×10^5^ M^−1^s^−1^ and increasing k_d_ from 0.021 s^−1^ to respectively 0.042, 0.227 and 0.066 s^−1^ (Figure 2B).

**Table 1:** Characteristics of the Nb_An33 mutants.

VHH name	Amino acid substitutions	k_a_ (M^−1^s^−1^)	k_d_ (s^−1^)	K_D_ (nM)	Trypanotoxic activity (%)
Nb_An33 [**24**]	Wildtype	7.41×10^5^	0.021	28	73
Nb_An33mut1	Ser29Arg Thr30Ala Tyr31Leu	4.67×10^5^	0.042	89	33
Nb_An33mut2	Val103Phe Arg104Val Ser105Asn Arg107Asn	3.14×10^5^	0.227	723	4
Nb_An33mut3	Val103Asn Arg104Asp Ser105Cys Arg107Leu	3.36×10^5^	0.066	197	24
Nb_An33hum1 [**18**]	Tyr37Val	2.43×10^5^	0.264	1100	0
Nb_An33hum2 [**18**]	Tyr37Val Glu44Gly Arg45Leu	2.91×10^5^	0.150	540	0
Nb_An33hum3 [**18**]	Glu44Gly Arg45Leu	7.02×10^5^	0.016	23.5	57

Wildtype Nb_An33 and the variants derived thereof with their respective amino acid substitutions, the affinity and kinetic rate constants and trypanotoxicity range as determined after 18 h incubation at 37°C with 20 µg/ml Nb.

In addition to these Nb_An33 variants, previously generated Nb_An33 humanized variants (Nb_An33hum1: Tyr37Val, Nb_An33hum2: Tyr37Val/Glu44Gly/Arg45Leu and Nb_An33hum3: Glu44Gly/Arg45Leu), mutated in the hallmark amino acids in framework-2 that distinguish VHHs from VHs (Tyr^37^, Glu^44^ and Arg^45^), were available for analysis of trypanotoxic potential [18]. Concerning their affinities, Tyr37 replacement drastically reduced the K_D_ from 28 nM to respectively 1.1 µM and 540 nM for Nb_An33hum1 and Nb_An33hum2 (Figure 2B, Table 1). Together with the reduction in affinity by mutagenesis of the Nb_An33 paratope and framework, trypanotoxic potential is strongly affected (Figure 2C, Table 1). Especially Nb_An33mut2, Nb_An33hum1 and Nb_An33hum2 are nearly entirely abrogated in trypanotoxic potential, related to the strongest reduction in affinity (respectively 723 nM, 1.1 µM and 540 nM) determined by slower on-rates (respectively 3.14×10^5^, 2.43×10^5^ and 2.91×10^5^ M^−1^s^−1^) and faster off-rates (respectively 0.227, 0.264 and 0.150 s^−1^) (Figure 2B, Table 1). Re-camelizing position 37 from Val to Tyr in the humanized variants (Nb_An33hum3) completely restores the K_D_ (23.5 nM), k_a_ (7.02×10^5^ M^−1^s^−1^), k_d_ (0.016 s^−1^) as well as trypanotoxic potential (Figure 2C, Table 1).

### Correlation between affinity and trypanotoxic potential of Nanobodies

**Figure 3:**
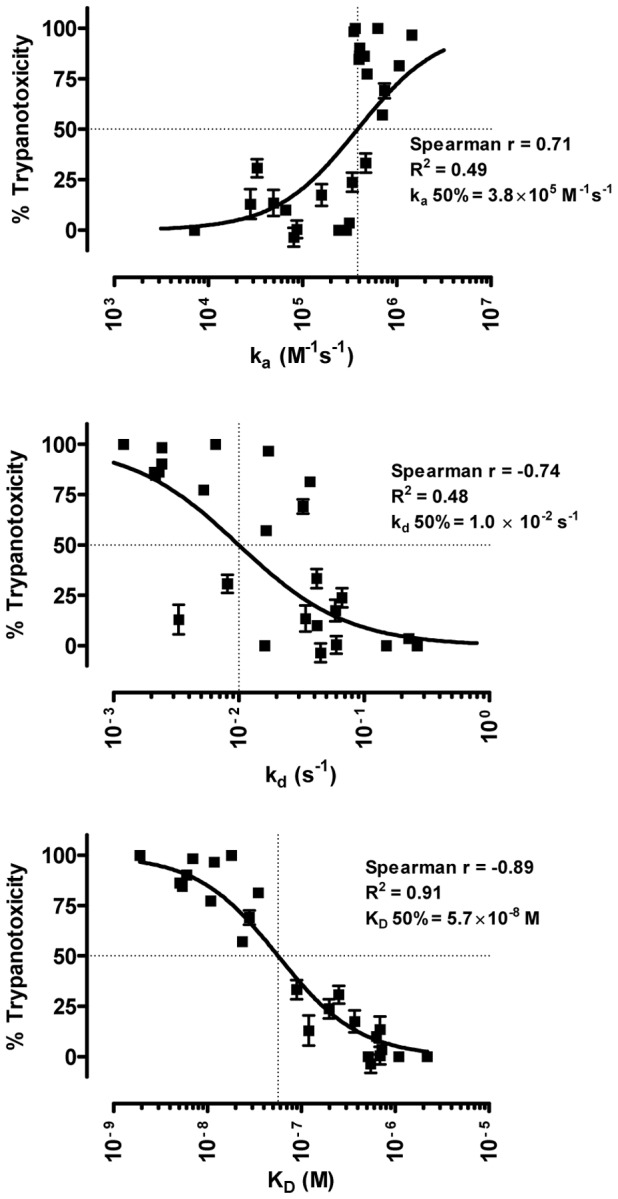
Analysis of the characteristics of the available panel of trypanotoxic Nanobodies. Non-linear three parameter regression and non-parametric Spearman correlation analysis of the trypanotoxic potential in function of k_a_, k_d_ and K_D_ respectively for all trypanotoxic Nbs (*n* = 25). R^2^ values, Spearman correlation coefficients (r) and half maximal (50%) effective kinetic constant values are indicated in each graph.

As a result of the identification of various AnTat1.1 sVSG-specific Nanobodies and the selection of Nb_An05 variants [16] and Nb_An33 variants, a set of Nanobodies (*n* = 25) with different trypanotoxic potential (0–100%) and affinity-range (2 nM–2.2 µM) became available. Within this set, four specificity groups could be identified by analyzing competitive binding of all Nanobody-combinations onto the CM5/VSG chip (Table 2). This indicates that each of these Nbs bind to one out of four different sites (epitopes) on the VSG. Plotting of trypanotoxic potential against the affinity and kinetic rate constants k_a_, k_d_ and K_D_, revealed a very good non-linear correlation of trypanotoxic potential with the K_D_ (R^2^ = 0.91), determined both by the kinetic rate association constant k_a_ (R^2^ = 0.48) and kinetic rate dissociation constant k_d_ (R^2^ = 0.47) (Figure 3). Spearman correlation analysis provided evidence for a strong correlation (p<0.0001) between the trypanotoxic activity and the k_a_ (Spearman correlation coefficient r = 0.71), k_d_ (r = −0.74) and especially K_D_ (r = −0.89). Half maximal effective (50%) affinity, k_a_ and k_d_ were calculated from the non-linear dose-response curves (Figure 3) yielding a K_D_ value of 57 nM (95% Confidence Interval (CI): 45–71), a k_a_ value of 3.8×10^5^ M^−1^s^−1^ (95% CI: 2.6×10^5^–5.6×10^5^) and a k_d_ value of 1.0×10^−2^ s^−1^ (95% CI: 6.3×10^−3^–1.6×10^−2^) that can be considered as critical cut-off values for anti-trypanosomal activity of Nbs. Under our experimental conditions (∼1.6 nM VSG, 1.3 µM Nb), we have calculated that Nbs with this critical K_D_ parameter would achieve a 95% saturation of the AnTat1.1 VSG molecules on the parasite coat.

**Table 2:** Characterization of the available panel of trypanotoxic Nanobodies.

Specificity group	VHH name	Affinity	Trypanotoxic activity
Group 1	Nb_An05 [**16**], Nb_AnR3.7, Nb_AnR3.92, Nb_AnR4.29, Nb_AnR4.11, Nb_AnR4.55, Nb_AnR4.71, Nb_AnR4.80	2–35 nM	80–100%
	Nb_An05mut2,4,5,6,9,12,17,19	120 nM–2.2 µM	0–35%
Group 2	Nb_An46 [**16**]	18 nM	100%
Group 3	Nb_AnR3.68	9 nM	>95%
Group 4	Nb_An33 [**24**], Nb_An33mut1-3, Nb_An33hum1-3 [**18**]	23 nM–1.1 µM	0–75%

Subdivision of the Nanobodies into 4 specificity groups by surface plasmon resonance determining cumulative or competitive binding onto the CM5/VSG-chip of all two-by-two Nanobody combinations. For each specificity group, the affinity range is indicated as well as the trypanotoxicity after 18 h incubation at 37°C with 20 µg/ml Nb.

## Discussion

The discovery of VHHs or Nanobodies with direct *in vitro* and *in vivo* trypanotoxic activity is a recent advancement in the development of new drug discovery approaches against African trypanosomiasis. The observed phenotype of lysis suggests that the VSG-specific Nbs interfere with the VSG recycling mechanism that - under normal conditions - is able to turn-over the total surface-exposed VSG pool within 12 minutes by endo/exocytosis in the flagellar pocket [13]. The previously identified Nanobodies, Nb_An05 and Nb_An46, were shown to lyse efficiently *T. b. brucei* parasites independent of the complement action, associated with a fast immobilization of the parasite and a pronounced swelling of the flagellar pocket [16]. Beside the physical stress of the enlarged flagellar pocket, an endocytosis/exocytosis malfunction might hamper the active and passive uptake of nutrients (as evidenced by using labeled transferrin, dextran and 2-deoxy-D-glucose) and result in cell death. Similar morphological phenotypes of lysis have been reported to occur upon RNAi mediated silencing of genes involved in clathrin-mediated endocytosis [26]–[29]. This indicates that Nanobodies target the endocytosis/exocytosis pathway but the exact mode-of-action has yet to be elucidated. It has been shown previously that the small size of the antigen-binding fragment is crucial, supported by the generation of trypanotoxic Fab′-fragments and the abrogation of trypanotoxic activity by addition of an Fc part to the VHH [16]. Suggesting that affinity is another important parameter, all previously and newly identified trypanotoxic Nanobodies have K_D_-values in the nanomolar range, a recurrent feature of Nanobodies that are selected by phage display from an immune VHH library [12], [25]. The association between affinity and trypanotoxicity was further supported by mutational analyses that were performed on Nb_An05, a highly trypanotoxic Nanobody [16] and Nb_An33, a trypanosome-specific Nanobody from which the structure has been resolved [18] and recognizes a cryptic and glycosylated (Man_9–5_GlcNAc_2_) epitope on *T. brucei* sVSG [24]. Using the highly trypanotoxic Nb_An05 as paradigm, we have illustrated previously that modification of the binding affinities by site-directed mutagenesis in the CDR regions resulted in strongly reduced trypanotoxic potential despite conservation of the capacity to bind to the trypanosome surface [16]. Here, we illustrate that the Nb_An33 exerts a limited trypanotoxicity over longer incubation times than those typically used for Nb_An05 and Nb_An46 [16]. This might be explained by the relatively lower affinity of Nb_An33 (28 nM) as compared to Nb_An05 (2 nM) and Nb_An46 (18 nM), although epitope-differences might also contribute to this difference. Variants in the Nb_An33 paratope (selected residues in the complementarity determining region 1 and 3) and in the framework (selected residues in framework-2 that differ between VHHs and human VHs [17], [18]) were generated respectively through a process of randomization by site directed mutagenesis and phage display. The selected variants exhibited lower affinities for their cognate antigen and displayed a significantly reduced or abrogated trypanotoxic activity. In the context of Nb_An33 humanization that was previously performed on framework region-2 [18], mutations that did not affect affinity, e.g. Glu44Gly/Arg45Leu, preserved the trypanotoxic activity as well. Moreover, Tyr37, a residue that is crucial for both conformational stability and antigen affinity [18], can not be substituted by the corresponding VH-residue valine without loss-of-activity as was observed for Nb_An33hum1 and Nb_An33hum2. Re-camelizing position 37 in Nb_An33hum2 (K_D_ = 540 nM) from valine to tyrosine in Nb_An33hum3 was sufficient to restore its affinity for VSG (K_D_ = 23.5 nM) as well as its trypanotoxic activity.

Incorporation of the affinity and trypanotoxicity data obtained from all available Nanobodies [16], [18], [24] confirmed a strong non-linear correlation between trypanotoxic activity and K_D_ (R^2^ = 0.91, Spearman correlation coefficient *r* = −0.89). This indicates that trypanotoxic activity requires a fast association (*r* = 0.71) and a slow dissociation (*r* = −0.74) of the bound Nanobody from the trypanosome surface. Non-linear dose-response analyses revealed apparent 50% activity at a K_D_ of 57 nM, a k_a_ of 3.8×10^5^ M^−1^s^−1^ and a k_d_ of 1.0×10^−2^ s^−1^. We have calculated that the 57 nM affinity (K_D_) yielding a 50% trypanotoxic activity under our experimental conditions would result in an occupancy of 95% saturation of all the VSG on the parasite surface coat by the Nb. As such, these data suggest that small molecule drug approaches against VSG would require surmounting a cut-off of 95% saturation of the antigen. The functional importance of affinity has previously also been documented for tumor cell lysis by complement activation upon binding of conventional antibodies to their antigen at the cell surface [30], [31]. In this case, the complement-dependent cytotoxicity was most efficient for monoclonal antibodies with slow off-rates and could be enhanced by reducing antibody dissociation using a F(ab′)_2_ cross-linking agent [30]. An analogous approach of generating multivalent constructs to decrease dissociation of Nanobodies from the trypanosome surface is unlikely to be successful given the cryptic nature of conserved epitopes buried in the dense variable antigenic VSG (variant-specific surface glycoprotein) coat [32]. Indeed, we have previously shown that bivalent Nb′_2_ are significantly less active than the monomeric forms [16]. In fact, the VSG-specificity of the toxic Nanobodies might impose a limitation on the drug discovery given that trypanosomes constantly alter VSG-epitopes in order to evade the host immune response [7], [8]. As such, there is the need to develop a Nanobody that can bind to an accessible, conserved epitope with sufficiently high affinity to achieve the required surface saturation.

As an alternative and future perspective, the procyclic developmental stage of the *T. brucei* parasite that occurs in the tsetse fly midgut displays a much smaller antigenic repertoire of glycosylphosphatidylinositol (GPI) anchored membrane proteins. Surface-binding lectins targeting N-glycans on procyclin were shown to be capable of inducing cell death in procyclic trypanosomes [33], [34]. Given the lower antigenic complexity of the procyclic antigenic coat and the previous illustration of an anti-trypanosomal activity upon binding to procyclin, this could represent an ideal target for Nbs. Therefore, we aim at generating Nbs against procyclin with kinetic parameters below the cut-off values as determined for the anti-blood stream form Nbs. Recently, a delivery system for functional Nanobodies to the tsetse fly based on its endosymbiont *Sodalis* has been developed [35], offering perspectives for anti-trypanosome Nbs to interfere with the trypanosome life cycle inside the insect vector.

### Accession numbers

AEI29603.1

AEI29601.1

AEI29599.1

AEI29604.1

AEI29602.1

AEI29600.1

AEI29598.1

AAP22642.1

AAP22644.1

## Supporting Information

Figure S1 **Aligned translated sequences of eight newly identified VHHs.** Eight novel Nbs selected by phage display and panning against *T. brucei* AnTat1.1 sVSG. Complementarity determining regions (CDR) 1–3 are indicated above the sequences.
(TIF)

Figure S2 **Aligned translated sequences of three novel Nb_An33 variants.** Three Nb_An33 variants (Nb_An33mut1-3 as compared to the wildtype Nb_An33) were randomized in selected positions in CDR1 or CDR3 and selected through phage display and panning. CDR1 to 3 are indicated above the sequence.
(TIF)
