# Correction: Affinity Is an Important Determinant of the Anti-Trypanosome Activity of Nanobodies

**DOI:** 10.1371/annotation/95386e26-78b2-44b1-af86-0028ba783156

**Published:** 2012-12-19

**Authors:** Guy Caljon, Benoît Stijlemans, Dirk Saerens, Jan Van Den Abbeele, Serge Muyldermans, Stefan Magez, Patrick De Baetselier

The image for Figure 1 is incorrect. Please view the correct image here:

http://www.plosntd.org/corrections/pntd.0001902.g001.cn.tif

