# Supplementary figures and images for: Affinity Is an Important Determinant of the Anti-Trypanosome Activity of Nanobodies
Source: PLoS Negl Trop Dis. 2012 Nov 15;6(11):e1902. doi: 10.1371/journal.pntd.0001902 (PMC3499403; doi:10.1371/journal.pntd.0001902)

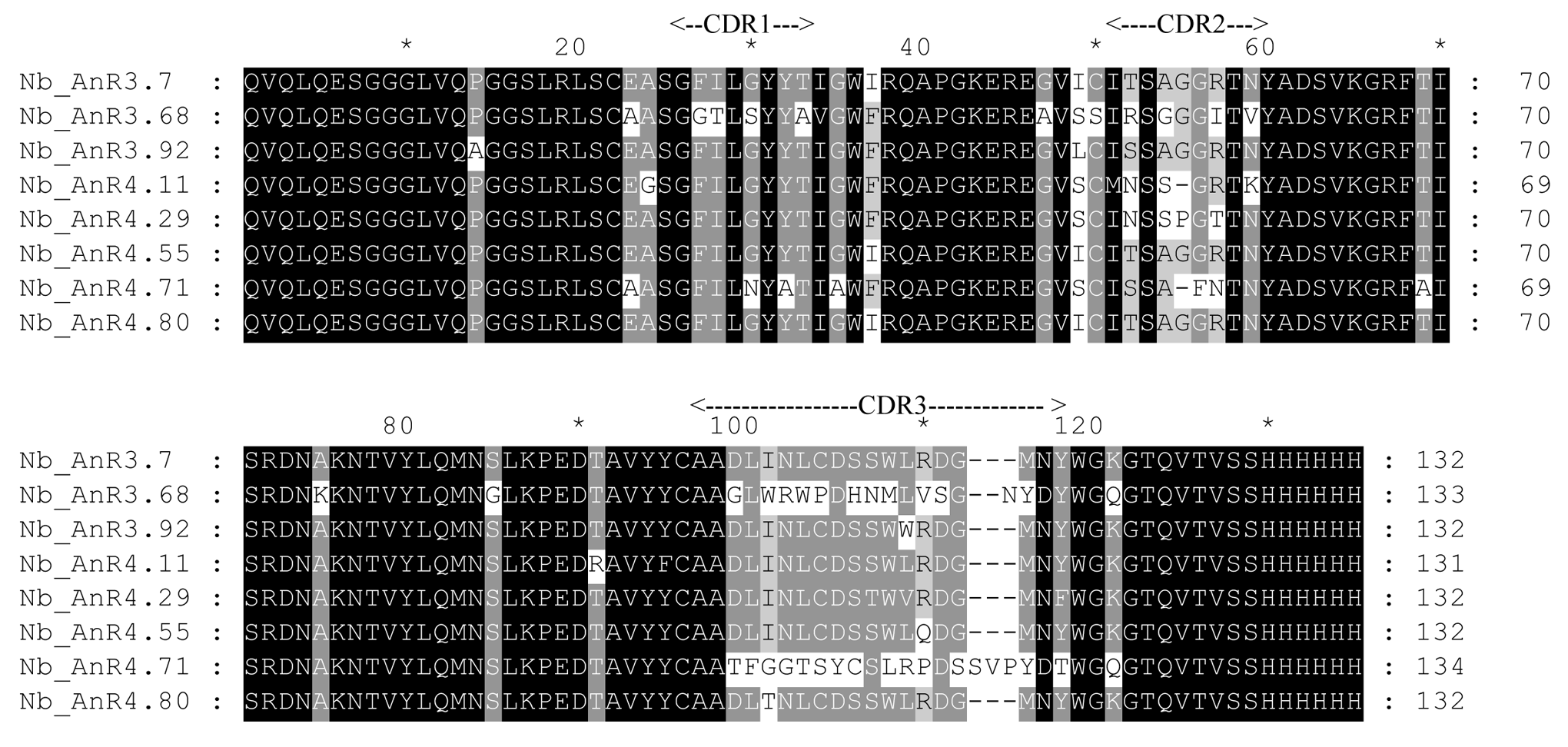

Supplement: Figure S1 — Aligned translated sequences of eight newly identified VHHs. Eight novel Nbs selected by phage display and panning against T. brucei AnTat1.1 sVSG. Complementarity determining regions (CDR) 1–3 are indicated above the sequences. (TIF) [file pntd.0001902.s001.tif]

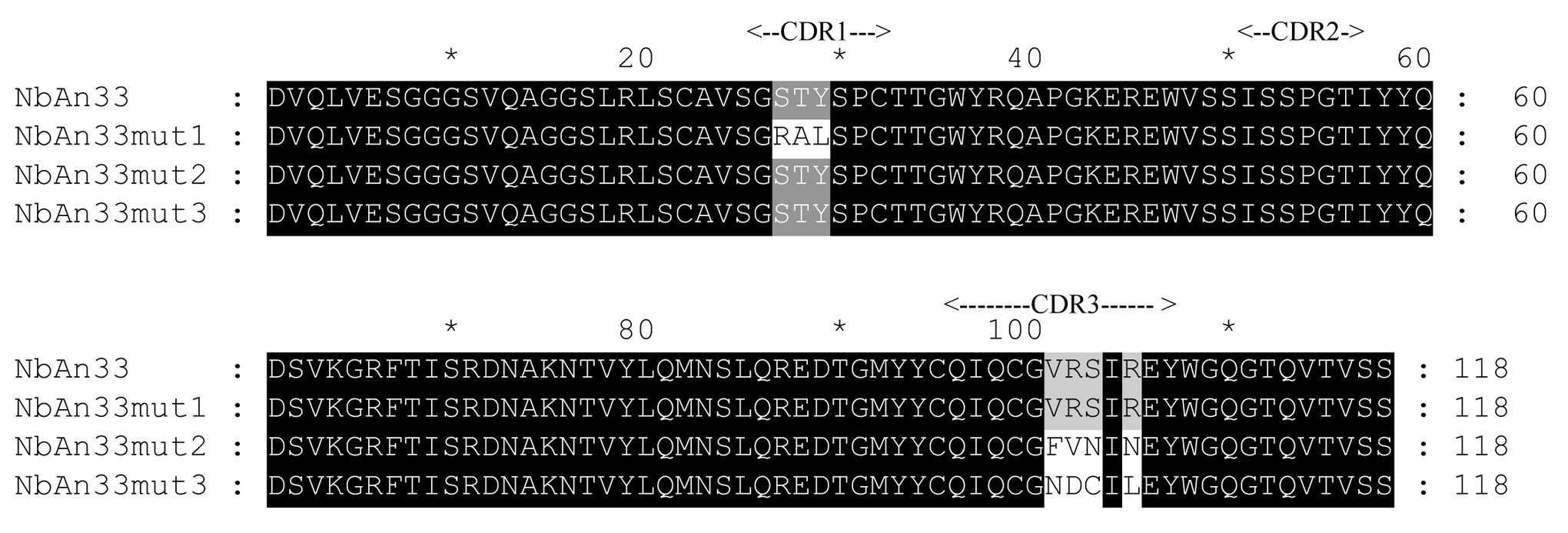

Supplement: Figure S2 — Aligned translated sequences of three novel Nb_An33 variants. Three Nb_An33 variants (Nb_An33mut1-3 as compared to the wildtype Nb_An33) were randomized in selected positions in CDR1 or CDR3 and selected through phage display and panning. CDR1 to 3 are indicated above the sequence. (TIF) [file pntd.0001902.s002.tif]
